# Analgesic Effect of Quadratus Lumborum Block Type III and Type II Versus Lateral Transversus Abdominis Plane Block in Cesarean Section: A Randomized Controlled Multicenter Trial

**DOI:** 10.5812/aapm-140464

**Published:** 2024-01-17

**Authors:** Hesham Elsayed Elashry, Mohamed Abdelbadie, Abeer Ali Elshabacy, Omnia Ali Elmiseery

**Affiliations:** 1 Department of Anesthesiology, Surgical Intensive Care and Pain Medicine, Faculty of Medicine, Tanta University, Tanta, Egypt; 2 Department of Anesthesiology, Surgical ICU and Pain Management, Faculty of Medicine, Beni-Suef University, Beni-Suef, Egypt; 3 Department of Obstetrics and Gynecology, Benha Teaching Hospital, General Authority for Hospitals and Teaching Institutes, Benha, Egypt

**Keywords:** Cesarean Section, Transversus Abdominis Plane Block, Quadratus Lumborum Block

## Abstract

**Background:**

Appropriate pain management promotes immediate mobilization and allows the parturient to adequately care for her neonate after cesarean section (CS).

**Objectives:**

This trial objective was to compare the type III and type II quadratus lumborum block (QLB) to transversus abdominis plane block (TAPB) regarding postoperative analgesic effect in CS.

**Methods:**

This randomized, controlled, single-blind trial involved 60 women presenting for CS under spinal anesthesia. The patients were assigned randomly to either the QLB type III, QLB type II, or lateral TAPB group. All blocks were performed using 20 mL of bupivacaine 0.25% bilaterally at the end of the operation with ultrasound guidance. Pain was assessed using the numerical rating scale (NRS) score at the post-anesthesia care unit (PACU) at 2, 4, 6, 8, 12, 18, and 24 hours. The level of patient satisfaction was graded on a 5-point Likert scale.

**Results:**

Numerical rating scale measurements at 6, 8, and 12 hours and total consumed meperidine in the 1st 24 hours after the operation were reduced significantly in QLB III than in QLB II and TAPB groups (P < 0.05) with an insignificant difference between the QLB II and TAPB groups (P > 0.05). The onset of the first request for analgesia was delayed significantly in QLB III, compared to QLB II and TAPB groups (P < 0.05), without a significant difference between the QLB II and TAPB groups (P > 0.05). Patient satisfaction and adverse events (e.g., postoperative nausea and vomiting, bradycardia, and hypotension) exhibited insignificant differences among the three groups (P > 0.05).

**Conclusions:**

The QLB type III ensured better analgesia as evidenced by significantly lower pain measurements and amount of meperidine in the first 24 hours after the operation with delayed time of the first rescue analgesia in comparison to QLB II and TAPB; however, QLB II and TAPB were similar.

## 1. Background

Cesarean section (CS) is accompanied by considerable postsurgical pain (1). Efficient pain control allows early mobilization and supports maternal care for the newborn. Although there are various drug options and administration routes, efficient and safe pain control methods after CS are still under investigation (2).

Commonly, opioids are used for post-CS pain relief. However, adverse events, such as nausea, vomiting, pruritus, and sedation, continue to be significant problems with opiates (3). In addition, opioid secretion during lactation continues to be a unique consideration after CS (4).

The transversus abdominis plane block (TAPB) is administered between the internal oblique muscle and the transversus abdominis muscle in the fascial plane, directly pointing to the somatic nerves T6 - L1 that run in this plane (5). In addition, meta-analyses demonstrate that it is an effective analgesic for somatic pain and diminishes opiate intake (6, 7). It has been reported that the TAPB is an efficient analgesic approach following CS (2).

The quadratus lumborum (QL) muscle block (QLB) is a fascial plane block as a local anesthetic (LA) is introduced nearby to the QL muscle to numb the thoracolumbar nerves. Quadratus lumborum block is categorized into four types according to drug administration location: I (lateral), II (posterior), III (anterior/transmuscular), and IV (intramuscular) (8-10). Quadratus lumborum block can effectively decrease both visceral and somatic pain by LA distribution to the thoracic paravertebral space (TPVS), as this block ensures effective pain control from the T7 to L1 dermatomes (11). Quadratus lumborum block is one of the regional techniques that provide the greatest benefit in post-CS pain control, as it is progressively applied in obstetric anesthetic practice to improve analgesic results (12-14).

The quadratus lumborum block is an easy-to-perform superficial fascial block between the posterior abdominal wall muscles (between QL and erector spinae) (15). With a simpler approach, the QL muscle separates the needle tip from the peritoneum, minimizing the risk of intraperitoneal perforation and bowel injury (9). The reason for the better analgesic effect of QLB type III than TAPB observed in the present trial might be due to the distribution of the drug along the thoracolumbar fascia and end of thoracic fascia into TPVS, which is covered with adipocytes and for which the local tissue blood supply is low, resulting in slow blood absorption of the LA agent (16). Additionally, QLB results in substantial sensory blocks, compared to TAPB (T10-L3 vs. T10-T12) (17).

In the QLB, LA spreads from its lumbar cranial deposition into the TPVS; this could explain why QLB seems able to improve the pain of somatic and visceral types and why QLB could pose an analgesic effect following abdominal procedures (18). Transversus abdominis plane block, on the other hand, involves penetration into the anterior abdominal wall and blocks only somatic fibers (19).

The quadratus lumborum block offered longer-lasting and more effective analgesia than the TAPB up to 72 hours after CS (16, 20-23). However, it has not yet been established which approach of QLB is superior to TAPB as the other QLB techniques for analgesia postoperatively in CS.

## 2. Objectives

This randomized single-blinded trial was carried out to compare the QLB type III and type II versus TAPB regarding efficacy in CS.

## 3. Methods

This randomized, controlled, single-blind trial incorporated 60 women aged 18 to 40 years, with American Society of Anesthesiologists (ASA) physical status II, presenting for elective CS in Tanta University hospitals and Benha Teaching Hospital in Egypt from March 2023 to June 2023. The study was carried out with the approval of the ethics committee of the general authority for teaching hospitals and institutes on 22/03/2023 (approval code: HB000132) and registration of clinicaltrials.gov (ID: NCT05950568). All cases provided signed, informed consent.

The exclusion criteria were body mass index (BMI) ≥ 40 kg/m^2^, weight < 50 kg, height < 150 cm, contraindications for the use of active labor, spinal anesthesia, recent opiate intake, hypersensitivity to any used medication, or substantial cardiovascular, renal, or hepatic diseases.

The cases were allocated randomly to either the QLB type III, QLB type II, or TAPB group on a 1: 1: 1 ratio in a parallel manner basis using a computer-generated randomization sequence. A piece of paper containing the procedure’s name was placed inside an envelope, and each envelope was assigned a number based on the arrays of numbers in the chart. The outcome assessor was blinded for group allocation.

Preoperative evaluation involves taking history, clinical examination, and routine laboratory tests. During the preoperative anesthesia visit, the participant was educated on the trial’s methodology and pain rating by the numerical rating scale (NRS).

Following cannula insertion, each patient received 500 mL of Ringer’s lactate solution in the operating room as a preload. Regular monitoring (e.g., temperature probe, noninvasive blood pressure, pulse oximetry, and 5-lead electrocardiogram [ECG]) were employed in this study. Cesarean section was performed under spinal anesthesia with 2 - 2.5 mL heavy bupivacaine 0.5% without adjuvants. Blocks were performed at the end of surgery by an anesthesiologist with comprehensive experience in ultrasound (US) guidance nerve block.

### 3.1. Quadratus Lumborum Block Technique

**Figure 1.**
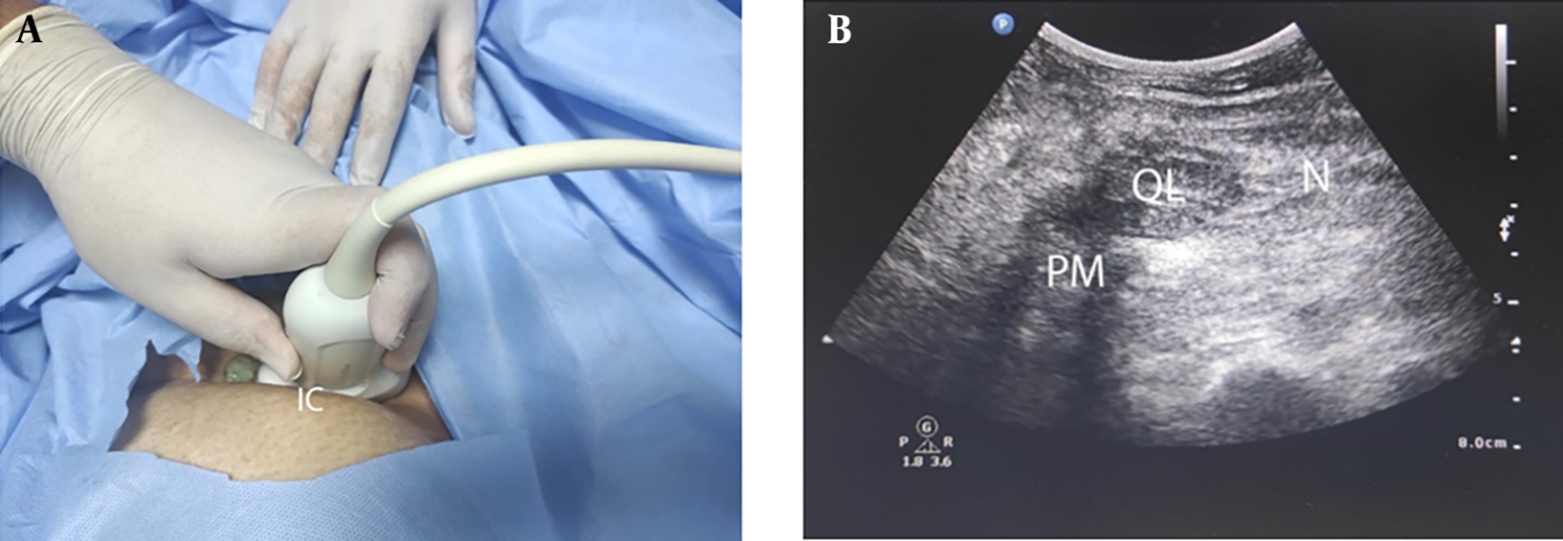
Quadratus lumborum block technique type III (A) position and (B) ultrasound image (IC, iliac crest; PM, psoas major; QL, quadratus lumborum; ES, erector spinae; LD, latissimus dorsi; TP, transverse process).

**Figure 2.**
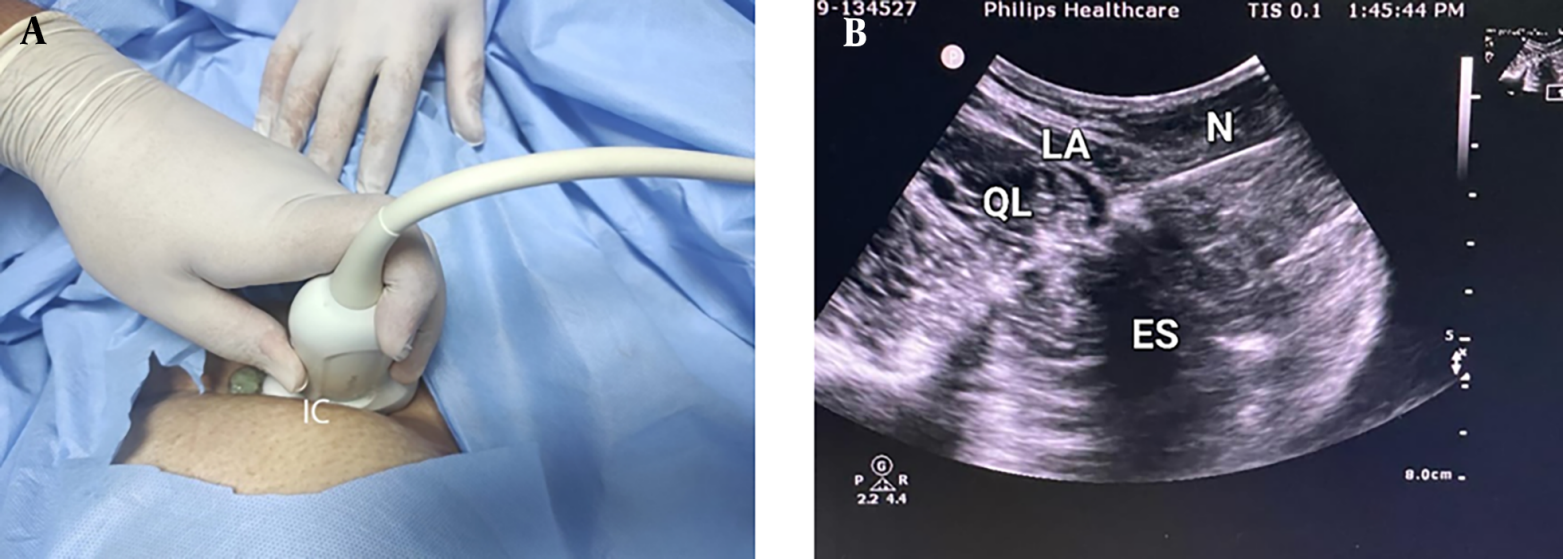
Quadratus lumborum block technique type II (A) position and (B) ultrasound image (IC, iliac crest; N, needle; QL, quadratus lumborum muscle).

Quadratus lumborum block was performed when the patient was positioned laterally decubitus. The low-frequency convex probe (Philips CX50 Extreme edition) was put in the flanks and moved to determine the transverse processes of the L2 or L3, the erector spinae, the psoas, and the QL muscles. A 22 G needle was advanced in the plane and positioned between the psoas and QL muscles at the anterior fascia lumbosacral in QLB III and between the QL muscle posterior border and the erector spinae muscle in the thoracolumbar fascia’s middle layer in QLB II. After negative aspiration, 2 mL of saline was administered to ascertain the needle's location. Hydro-dissection of the injectate at a location of concern of lumbosacral fascia and surrounding structure was depicted in real-time. Each side was then injected with 20 mL of bupivacaine 0.25% (Figures 1 and 2). 

### 3.2. Transversus Abdominis Plane Block Technique

**Figure 3.**
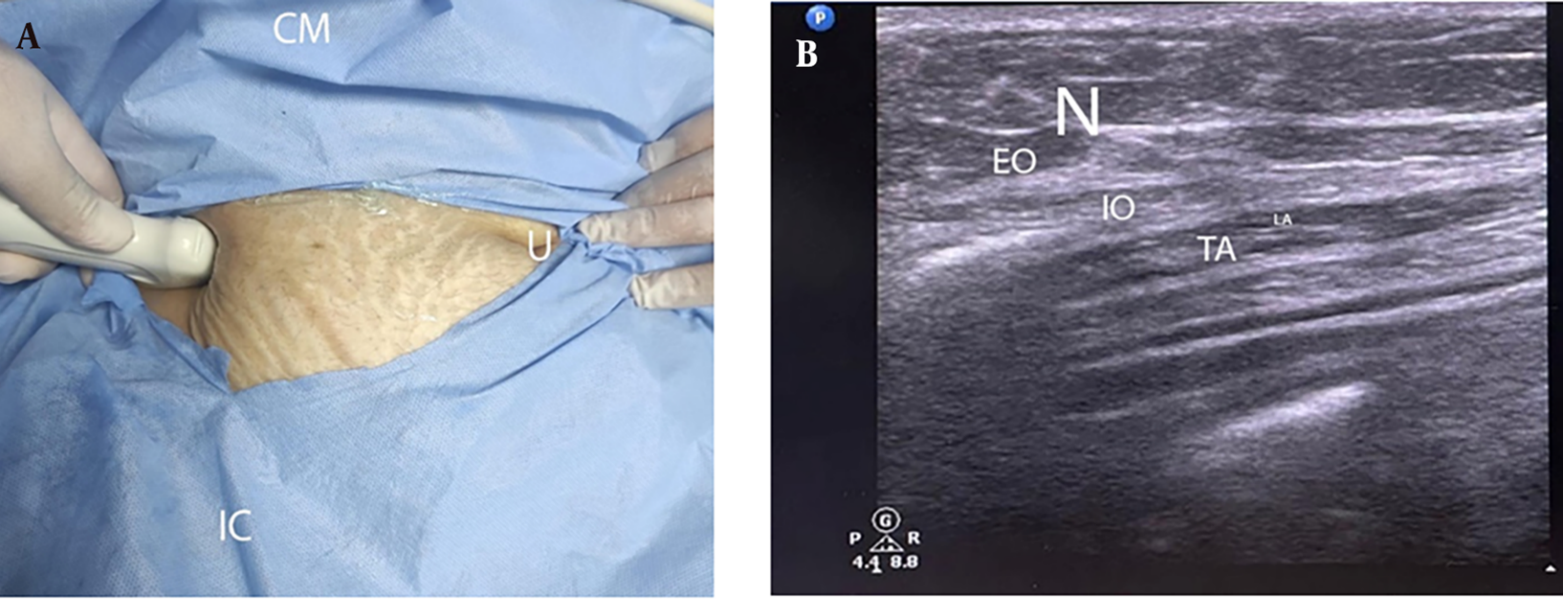
Transversus abdominis plane block technique (A) position and (B) ultrasound image (CM, costal margin; IC, iliac crest; U, umbilicus; N, needle; EO, external oblique muscle; IO, internal oblique muscle; TA, transversus abdominis muscle; LA, local anesthetic).

Transversus abdominis plane block was performed when the patient was positioned in the supine decubitus. A linear high-frequency transducer (Philips CX50 Extreme edition) was placed transversely on the anterolateral abdominal wall between the iliac crest and the costal margin. Under US guidance, the three layers of muscles, the external oblique, the internal oblique, and the transversus abdominis, were identified. A 22-gauge, 100-mm needle was then injected through the skin anteriorly in the plane and advanced into the fascial plane between the internal oblique and transversus abdominis muscles, with its tip lying in the mid-axillary line. To assist with identifying these structures, the probe was moved anteriorly to the rectus sheath, and the fascial planes followed laterally. Following negative aspiration, 2 mL of saline was injected to ascertain the needle location upon the visualization of the transversus abdominis. Each side was then implanted with 20 mL of bupivacaine 0.25% (Figure 3). 

### 3.3. Postoperative Management

Postoperative heart rate (HR) and mean arterial blood pressure (MAP) were reported at the post-anesthesia care unit (PACU) at 2, 4, 6, 8, 12, 18, and 24 hours. All cases received paracetamol 15 mg/kg infusion/6 h as routine analgesia. The postoperative NRS score was evaluated at the PACU at 2, 4, 6, 8, 12, 18, and 24 hours. If the NRS score remained ≥ 4, a bolus of meperidine (0.5 mg/kg intravenously [IV]) was provided and repeated after 30 minutes if NRS remains ≥ 4. The onset of the first analgesia rescue and the total amount of meperidine in 1st 24 hours after the operation were also recorded.

The level of patient satisfaction was graded on a 5-point Likert scale (24) (0 = extremely dissatisfied, 1 = dissatisfied, 2 = neither satisfied nor dissatisfied, 3 = satisfied, 4 = extremely satisfied). Side effects, such as hypotension (defined as any decrease in the MAP of > 20% of the preoperative baseline value or MAP ≤ 65 mmHg), bradycardia (HR < 60 beats/min), and postoperative nausea and vomiting (PONV), were documented. Hypotension was managed with fluid bolus ± ephedrine 5 - 10 mg IV. Bradycardia was managed with 0.01 mg/kg of atropine. The prevention of PONV was attempted using 4 mg ondansetron at the end of the operation.

The primary outcome was the total postoperative consumed meperidine in the first 24 hours. The secondary outcomes were the time of the first analgesic request, postoperative pain, and patient satisfaction.

### 3.4. Sample Size Calculation

The sample size calculation was made by G*Power 3.1.9.2 (Universitat Kiel, Germany). A pilot study was performed (5 cases per group), and it was observed that the mean (± standard deviation [SD]) of the total consumption of meperidine postoperatively in the first 24 hours was 48 ± 16.81, 76 ± 36.98, and 84 ± 23.02 mg in QLB III, QLB II, and TAPB groups, respectively. In this study, 20 women were recruited in each group based on a 0.6 effect size, 95% confidence limit, 95% study power, and 1: 1: 1 group ratio. Moreover, four cases were added to each group (to compensate for dropout).

### 3.5. Statistical Analysis

In this study, SPSS software (version 27; IBM, Chicago, IL, USA) was used for statistical analysis. The normality of the data distribution was verified by the Shapiro-Wilk test and histograms. Parametric quantitative data were expressed as mean and SD and assessed using the analysis of variance (ANOVA) (F) test with post hoc comparisons (Tukey) and repeated measures ANOVA. Quantitative non-parametric data were expressed as the median and interquartile range (IQR) and compared between groups using the Kruskal-Wallis test (with the Mann-Whitney test for pairwise comparison). The chi-square test was applied to analyze qualitative variables that were presented as frequency and percentage (%). A two-tailed P-value ≤ 0.05 was judged statistically significant.

## 4. Results

**Figure 4.**
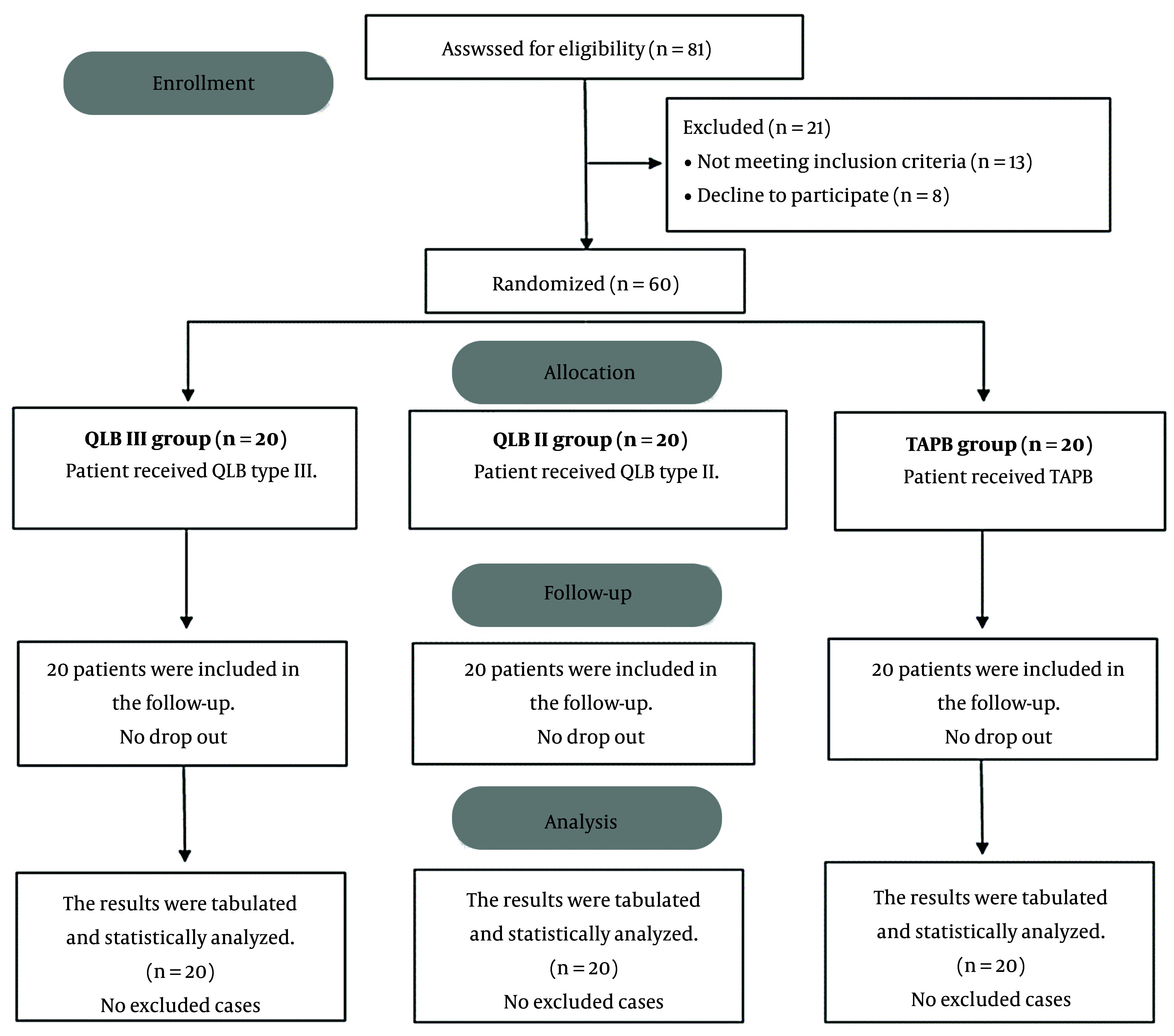
CONSORT flowchart of the enrolled patients

In this study, 81 cases were evaluated for eligibility, and 21 cases were excluded. The remaining cases were allocated randomly to three groups (20 patients each). All allocated cases were followed up and statistically analyzed (Figure 4). 

Demographic information and duration of surgery were comparable between the studied groups (Table 1). 

**Table 1.** Demographic Data and Surgery Duration of the Studied Groups ^a^

Variables	QLB III Group (n = 20)	QLB II Group (n = 20)	TAPB Group (n = 20)	P-Value
**Age, y**	28.2 ± 6.41	30.2 ± 4.98	31.7 ± 5.84	0.159
**Weight, kg**	73.6 ± 7.35	76.4 ± 7.18	74.9 ± 5.52	0.425
**Height, m**	1.7 ± 0.08	1.7 ± 0.08	1.7 ± 0.06	0.641
**BMI, kg/m** ^**2**^	26.7 ± 3.53	27.1 ± 4.31	26.6 ± 2.41	0.873
**Duration of surgery, min**	43 ± 4.41	43.3 ± 5.2	46.3 ± 7.59	0.161

Abbreviations: QLB, quadratus lumborum block; TAPB, transversus abdominis plane block; BMI, body mass index; ASA, American Society of Anesthesiologists.

^a^ Values are expressed as mean ± standard deviation (SD).

**Figure 5.**
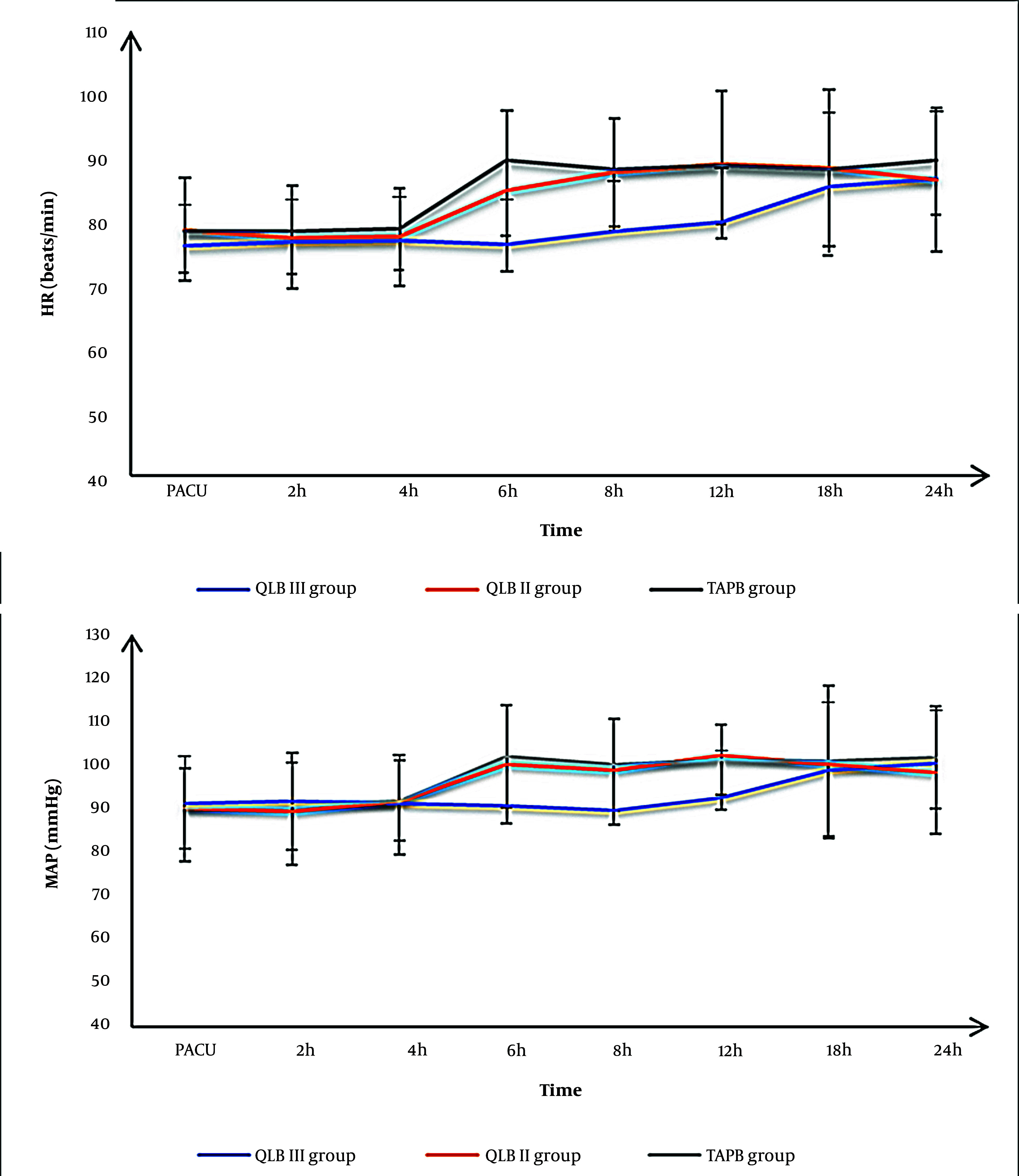
Heart rate (HR) (A) and mean arterial blood pressure (MAP) (B) of the studied groups (PACU, post-anesthesia care unit).

Postoperative HR and MAP measurements at the PACU at 2, 4, 18, and 24 hours were matched among the three groups; however, at 6, 8, and 12 hours were lower significantly in the QLB III group than in the QLB II group and TAPB group (P < 0.05) without a significant difference between QLB II and TAPB groups (Figure 5). 

The numerical rating scale measurements at the PACU at 2, 4, 18, and 24 hours were comparable among the three groups; however, at 6, 8, and 12 hours were lower significantly in the QLB III group than in the QLB II group and TAPB group (P < 0.05) without a significant difference between QLB II and TAPB groups (Table 2). 

**Table 2.** The Numerical Rating Scale (NRS) of the Studied Groups ^a^

Variables	QLB III Group (n = 20)	QLB II Group (n = 20)	TAPB Group (n = 20)	P-Value	Post hoc ^b^		
					P1	P2	P3
**PACU**	0 (0 - 1)	0 (0 - 1)	1 (0 - 1)	0.249			
**2 h**	1 (0 - 1)	0 (0 - 1)	1 (0 - 1)	0.130			
**4 h**	1 (0 - 1)	1 (1 - 1.25)	1 (1 - 2)	0.160			
**6 h**	1 (1 - 2)	2 (2 - 6)	3.5 (2 - 4.25)	< 0.001	< 0.001	< 0.001	0.795
**8 h**	2 (1.75 - 2)	3 (2.75 - 3.5)	3 (2 - 4)	0.003	0.002	0.005	0.760
**12 h**	1.5 (1 - 4)	4.5 (2.75 - 6)	3 (2.75 - 5)	< 0.001	< 0.001	0.004	0.443
**18 h**	2 (1 - 4)	4 (2 - 6)	3.5 (2 - 5)	0.145			
**24 h**	4 (1 - 4)	3.5 (2.75 - 5)	4 (2 - 5)	0.578			

Abbreviations: QLB, quadratus lumborum block; TAPB, transversus abdominis plane block.

^a^ Values are expressed as median (interquartile range [IQR]).

^b^ P1: P-value between QLB III group and QLB II group; P2: P-value between QLB III group and TAPB group; P3: P-value between QLB II group and TAPB group.

The mean ± SD of the first analgesia request time was 12.9 ± 3.21, 7.1 ± 1.1, and 7.4 ± 1.6 hours in the QLB III, QLB II, and TAPB groups, respectively. The mean ± SD of the total amount of meperidine in the first 24 hours after the operation was 77.6 ± 15.96, 99.3 ± 27.8, and 91.4 ± 18.57 mg in the QLB III, QLB II, and TAPB groups, respectively. The time of the first analgesia request was significantly delayed in the QLB III group in comparison to the QLB II and TAPB groups (P < 0.001), with an insignificant difference between the QLB II and TAPB groups. The total amount of meperidine in the first 24 hours after the operation was significantly lower in the QLB III group than in the QLB II and TAPB groups (P < 0.001), with an insignificant difference between the QLB II and TAPB groups (Table 3). Patient satisfaction and adverse events (e.g., hypotension, bradycardia, and PONV) were insignificantly different among the three groups (Table 3). 

**Table 3.** Time of the First Rescue Analgesia, the Total Amount of Meperidine in the First 24 Hours After the Operation, Patient Satisfaction, and Adverse Events of Studied Groups ^a^

Variables	QLB III Group (n = 20)	QLB II Group (n = 20)	TAPB Group (n = 20)	P-Value	Post hoc ^b^		
					P1	P2	P3
**Time of first rescue analgesia, h**	12.9 ± 3.21	7.1 ± 1.1	7.4 ± 1.6	< 0.001	< 0.001	< 0.001	0.726
**The total amount of meperidine in the first 24 hours postoperative, mg**	77.6 ± 15.96	99.3 ± 27.8	91.4 ± 18.57	< 0.001	< 0.001	< 0.001	0.866
**Patient satisfaction**							
Very satisfied	18 (90)	14 (70)	13 (65)	0.155			
Satisfied	2 (10)	6 (30)	7 (35)				
**Adverse events**							
Hypotension	3 (15)	5 (25)	4 (20)	0.732			
Bradycardia	2 (10)	3 (15)	4 (20)	0.676			
PONV	3 (15)	4 (20)	6 (30)	0.503			

Abbreviations: QLB, quadratus lumborum block; TAPB, transversus abdominis plane block; PONV, postoperative nausea and vomiting.

^a^ Values are expressed as mean ± standard deviation (SD) or No. (%).

^b^ P1: P-value between QLB III group and QLB II group; P2: P-value between QLB III group and TAPB group; P3: P-value between QLB II group and TAPB group.

## 5. Discussion

Regional anesthesia is a promising approach for pain control after CS because it facilitates early mobility and breastfeeding deprived of opioid-associated side effects (25, 26). According to the results of the present study, QLB III ensured better analgesia as evidenced by NRS measurements at 6, 8, and 12 hours, and the total consumed meperidine in the first 24 hours after the operation was significantly lower in the QLB III than in the QLB II and TAPB groups without significant differences between the QLB II and TAPB groups. The time of the first rescue for analgesia was delayed significantly in the QLB III group in comparison to the QLB II and TAPB groups, without a significant difference between the QLB II and TAPB groups.

Some investigations have demonstrated that the QLB could ensure a superior analgesic effect to the TAPB in cases undergoing CS (23, 27).

To the best of our knowledge, this is the first study that compares the two approaches of QLB (II and III) to TAPB in CS. The latest systematic review and meta-analysis by Liu et al. (28) revealed that after abdominal surgery, the QLB ensured excellent pain control with less consumption of opiates than the TAPB. In addition, the QLB offered longer-lasting and more effective analgesia than the TAPB up to 72 hours after CS (16, 20-23). Recently, the QLB was much more effective than TAPB after CS (29, 30).

Regarding comparing the two QLB approaches, the results of the present study are in line with the results of Megawer et al., who reported that QLB type III provided significantly better analgesia than type II after CS (31). This finding could be justified as LA spread more deeply into tissues in QLB III.

In a separate study involving volunteers, QLB III was administered 1 hour after a magnetic resonance (MR) examination. The conclusions demonstrated the injection distribution through the QL and psoas muscles in a cranial direction (32). Additionally, Koksal et al. (33) reported that consumed morphine reduced significantly in the QLB-III than in the QLB-II group. The QLB-III group showed reduced considerably resting pain scores after CS.

Moreover, in a previous study, QLB III offered more efficient, longer-duration analgesia postoperatively and reduced opiate consumption than QLB II in cases undergoing repair of an open inguinal hernia under spinal anesthesia (34). Furthermore, it was observed that the consumption of morphine and pain scores reduced significantly in QLB III than in QLB II after CS (35). The results of the current study revealed that patient satisfaction and adverse events insignificantly differed among the three groups. Similar findings were obtained in other studies (20, 31).

### 5.1. Limitations

The sample size was relatively small to prove the secondary outcomes. The cases were followed up for a relatively short duration as pain after CS might continue for more than 24 hours; accordingly, a longer monitoring duration is important (6). Therefore, large-scale studies with a longer follow-up duration are needed to generalize the findings of the present study.

### 5.2. Conclusions

Quadratus lumborum block III ensured better analgesia as evidenced by significantly lower pain measurements and amount of meperidine in the first 24 hours after the operation with delayed time of first rescue analgesia in the QLB III group in comparison to the QLB II and TAPB groups; however, QLB II group and TAPB group were comparable.

## Data Availability

The dataset presented in the study is available on request from the corresponding author during submission or after publication.

## Clinical Trial Registration Code:

NCT05950568.
